# Supplementary material for: Height & income: Labor returns of health in Mexico from 2000 to 2018
Source: PLoS One. 2024 May 14;19(5):e0303108. doi: 10.1371/journal.pone.0303108 (PMC11093369; doi:10.1371/journal.pone.0303108)
Supplement: Attachment — Submitted filename: Answers to comments.docx [file pone.0303108.s001.docx]

| Comment | Answer/action |
| --- | --- |
| Reviewer 1 |  |
| 1. Are the 4 surveys comparable? I think that the questionnaire of the 2000 survey is different, and I am not sure if it is comparable to the others. | Sampling framework is equivalent for the 4 surveys, and the questionnaires from each round are based on those from the previous surveys. While there is some evolution of the questionnaires over time, for the variables used in this analysis, the questions are comparable. |
| 2. Are the anthropometric measures taken by qualified personnel or self-reported by respondents. Please clarify. | Anthropometric measures were taken by qualified personnel; we added a sentence to clarify this. |
| 3. It seems that it is a coauthored paper, but in some sentences, it is referred as, for example: “In addition, I generated a socioeconomic status index of the household…” | We corrected this, thanks. |
| 4. The authors use individuals between 25-45 y/o to focus on those whose height is the maximum possible and has not yet beginning to decline. I think this selection needs a reference. Please add a reference. | References included now. |
| 5. Do you [have] individuals with income equal to zero in your sample? If so, how do you treat this cases when applying the natural logarithm? | No, surveys collected data on remunerated labor, so most cases reported a positive income, and for the other cases data on income were coded as missing. The later cases are captured in the Heckman model which can accommodate missing values. |
| 6. Analysis section: “We estimated experience as age minus the schooling years and minus 6 years, as suggested in previous studies”, please specify which studies. Add the references. | Reference included now. Nevertheless, we removed this variable from the estimations, as it was causing multicollinearity. |
| 7. The results for the education coefficient are somehow surprising (sometimes negative). The authors only describe the results but do not provide a possible explanation or discussion. I think a discussion on this is needed. | After we removed the variable experience from the estimations, the coefficients for schooling are now consistent. |
| Reviewer 2 |  |
| Abstract:  This sentence needs to be revised using adults' height as an indicator of the individual's health capital accumulation. Previous analyses have reported inconsistent estimates of the health returns in Mexico, although they use different approaches. Which approaches? And compared to what?  In addition, the purpose of the paper in the abstract is not clear. | Abstract was edited in response to the suggestions |
| Introduction:  The introduction should be written in the third person. | We prefer not to use the third person and note that both are common in this journal. If the editors would like us to change the abstract to the third person, we can do so. |
| Methodology:  It should clearly state the data sources, the sampling method of each data source, and the sample size. | We added additional detail on the surveys, sampling procedures and questionnaires. |
| Also, it is important to clearly present the methods of generating variables such as income, it is not clear if it was an imputation or if the authors created a wealth index. This information should be provided in the text; if necessary, additional information should be included as supplementary material. | As we described in the manuscript, data on earnings is self-reported at the household, there is no imputation for this variable |
| Please use subheadings to present the data, variables and methods. | Added |
| It Will be ideal that the authors present a more detailed description of the different methods, and how they harmonized the data to make all surveys comparable across years. | Models were estimated for each year separately; as the surveys are implemented by the same institution, questionnaires and measurements are similar. In particular, the variables included in the analysis were collected using similar questions. |
| Results  It is necessary that authros explain better why the percentage of men across time has reduced. Most surveys collect information from all household members, not only from the person who is at the house. Therefore, depending on the data collection process, the survey Will or may not be representative of individuals. A SEntence such as "is common to have a larger percentage of women as they are more likely to be at their dwellings, a fact that is confirmed by the differential in labor participation" only applys if the survey only collects information from one main respondent and it does not collect information of other household members. Please explain this better, because for example ENSANUT is representative of members of private households, therefore men and women (regardless of whether they are at home when the survey was collected). | We agree with the reviewer that the percentage of men in the survey is significantly higher in 2000 than in the subsequent survey years. We don’t have an explanation for that difference but suspect that it does not reflect a demographic change but rather something that was specific to the timing or the way that the survey was conducted in 2000. We clarified in the manuscript that some data can be collected about a member who is not present, but that anthropometric measurements can only be taken if the individual is present. |
| If the sample between 2000 and 2006 was not comparable, why did you only see this on the education indicators? It is necessary to explain this better and include references supporting the argument that the oversampling of poor areas did not correct sampling weights. | Thanks; the argument is the other way around: the sampling weights used did not fully correct for the oversampling. We tried to clarify this in the revised manuscript. |
| Table 1 needs to be presented better; in its current form, it is confusing and not easy to read. Also percentages should be presented as percentages not as a fractions. | We updated the table as suggested. |
| Can you please include CI in figure 1. Also it will be better if you include in the X axis the years, and each of the lines represents a decile, in that way it is possible to see the gap better.  Also, please consider that line graphs are not used to present decides. In that case use bar graphs | A new version of figure 1 is included in the revised version. |
| Table 2 is confusing and needs to be presented better, | We updated the table as suggested |
| Authors need to explain better why the education variable behaves so differently between models and what are the implications of this, given that education is a stronger explanatory variable, this should be explained in detail and a more comprehensive analysis should be conducted. | Please see the response to the reviewer 1 on this. |
| One main problem that I find in the article is that the authors give the sense that an increase in height increases income, but there is not a significant increase in height over time on average, and income does not increase either. In addition, the article does not provide enough details to understand if the increase in height for people in the highest decile was larger than in the lowest decile, or at least how the data is presented it is not possible to understand if the differences are significant and increase over time. Most importantly, the data is not comparable over time, or at least this is not clear in the methods section (does 2000 data uses the same sampling frame as 2018?) therefore, it is not clear if the analysis can be conducted with this data, and if the results are showing real differences over time, or just differences in the sampling frame. | Sampling frames are equivalent for the four surveys (we clarify this in the revised manuscript), so, are comparable. While averages are similar over tims, the argument for mobility is not about the average but rather at the individual level: gains in height are associated with gains in income holding everything else constant. |
| Finally, the results of the four estimation models are not consistent; this is not properly explained in the article.  The discussion does not present limitations and the discussion needs to be improved, including potential explanations for the results. | We have expanded the previous discussion of limitations. |
| The changes in the education results for men and women are difficult to explain. | It is not clear what changes the reviewer is referring. We edited as referred in the previous comment on the effects of schooling, and expanded the discussion related to this. |
| The article needs to improve the way tables and graphs are presented. | Revised |
| Finally the article should be copy-edited. | We have extensively copy-edited the manuscript. |
