# Supplementary material for: Height & income: Labor returns of health in Mexico from 2000 to 2018
Source: PLoS One. 2024 May 14;19(5):e0303108. doi: 10.1371/journal.pone.0303108 (PMC11093369; doi:10.1371/journal.pone.0303108)
Supplement: Attachment — Submitted filename: Response to comments 15Apr2024.docx [file pone.0303108.s002.docx]

| Comment | Response |
| --- | --- |
| Editorial |  |
| Please review your reference list to ensure that it is complete and correct. If you have cited papers that have been retracted, please include the rationale for doing so in the manuscript text, or remove these references and replace them with relevant current references. Any changes to the reference list should be mentioned in the rebuttal letter that accompanies your revised manuscript. If you need to cite a retracted article, indicate the article’s retracted status in the References list and also include a citation and full reference for the retraction notice | No retracted manuscripts are cited. We updated the references list to the Plos style (we are using EndNote). |
| Reviewer #2: |  |
| Please edit and present better the tables, in the current state is really difficult to follow and understand. | We edited the tables to 2 decimals |
| Also, please check that you the evidence you are presenting does not support the ideat that "are there is a robust positive relationship between height and socioeconomic decile.", only in extreme cases there are significant differences between hight. | This comment appears to be based on figure 1; except for 2000, there is a clear trend in height from the lower to the higher decile. We deleted the word robust from this sentence to avoid confusion. |
| Also explian and present the robustness analysiss. | As mentioned, as this only the descriptive part, we deleted the word robust here. |
| Finally, you are presenting three models, each reach to a similar conclusion, but still in some models the coefficient of years of schooling is not expected (e.g. 2012) and the difference between the models vs the IV models is large, please explain why and which model is the best | We appreciate the opportunity to clarify the implications of the different models presented in our study. It is important to emphasize that the designation of a "best" model is contingent upon the underlying assumptions and the context of the application. The variation in coefficients, can indeed be attributed to the potential endogeneity that the models without instrumental variables (IV) may not adequately adjust for. This endogeneity is precisely what our IV approach aims to correct.  The coefficients derived from models without IV tend to be lower, likely due to unaddressed endogeneity. On the other hand, if one accepts the validity of our proposed instrument — which we argue for rigorously — then the models utilizing this instrument offer estimates that we believe are more reflective of the true returns of height.  To address your concern, we have augmented the discussion section with an additional sentence to elucidate this point.  As for the unexpected result for education among females in 2012, we included a potential explanation. |
| Did you conduct any comparison between the thre models | We are not sure what type of comparison the reviewer is implicitly suggesting. All of the models are intended to estimate returns; we compare the coefficients between the different models. |
